# An Insight Into the Bacteriological Profile: Escalating Antimicrobial Resistance Patterns and the Spectrum of Multidrug-Resistant Enterobacteriaceae at a Tertiary Care Center in Western Maharashtra

**DOI:** 10.7759/cureus.69538

**Published:** 2024-09-16

**Authors:** Shanu Sharma, Geeta Karande, Satish Patil

**Affiliations:** 1 Department of Microbiology, Krishna Institute of Medical Sciences, Krishna Vishwa Vidyapeeth (Deemed to be University), Satara, IND

**Keywords:** clinical and laboratory standards institute, enterobacteriaceae, extended spectrum β-lactamases, metallo β-lactamases, multidrug resistant

## Abstract

Background: Enterobacteriaceae are a group of aerobic and facultative anaerobic Gram-negative bacilli known to cause various infections in healthy folks and those with preexisting health conditions.

Aim: Current research focuses on analyzing the bacteriological profile of clinical isolates, examining their antimicrobial susceptibility and the spectrum of drug resistance.

Materials and methods: The study was conducted on patients admitted to the inpatient/outpatient department at Krishna Hospital, Karad. Clinical samples from patients with suspected infections, including pus, sputum, urine, blood, and other body fluids, were examined. The 156 bacterial isolates were identified using Gram staining and biochemical tests following the standard protocol.

Results: Of the 156 isolates, *Klebsiella pneumoniae *was the most common isolate, with 79 (50.6%), followed by *Escherichia coli*, with 52 (33.3%). The maximum resistance was demonstrated toward co-trimoxazole (96.3%), ciprofloxacin (88.7%), and ceftazidime (84.6%).

Conclusion: During the study, the prevalence of metallo-β-lactamase (MBL), extended-spectrum β-lactamase (ESBL), and AmpC increased significantly. The study data showed the overall prevalence of MBL, ESBL, and AmpC to be 67.3%, 37.8%, and 38.4%, respectively. Further, the rate of multidrug-resistant isolates was noteworthy (92.6%). Thus, comprehending the resistance pattern and epidemiology of the organism within a specific demographic area can help create better guidelines to curtail such infections.

## Introduction

Enterobacteriaceae are characterized as gram-negative bacilli measuring 1-3 μm in length. They exhibit facultative anaerobic metabolism and are positive for catalase activity. These bacteria demonstrate growth on MacConkey agar, a medium selective for gram-negative organisms based on their ability to ferment lactose. Enterobacteriaceae are indigenous to the intestinal tract of humans and animals, where they reside as commensal organisms [1].

The family Enterobacteriaceae encompasses 44 genera and approximately 176 species. Classification within this family is based on lactose fermentation, dividing members into three groups: 1) lactose fermenters, including *Escherichia coli*,*Enterobacter* spp., and *Klebsiella* spp.; 2) late lactose fermenters, such as *Citrobacter* spp. and *Serratia* spp.; and 3) non-lactose fermenters, which encompass *Edwardsiella*,* Hafnia*, *Morganella morganii*, *Proteus* spp., *Providencia* spp., *Salmonella* spp., *Shigella* spp., and *Yersinia* spp. [2]. With the advent of molecular methods, taxonomic classifications have evolved. In 2016, the order Enterobacterales was prioritized, leading to the proposal of several new families within this order. Members of the Enterobacteriaceae family are prominent pathogens implicated in a wide array of healthcare-associated infections [3]. The resistance of gram-negative bacteria to various antibiotics has markedly risen in recent decades, emerging as a critical global issue [4]. A significant mechanism contributing to this resistance involves the production of enzymes that degrade β-lactam antibiotics. Metallo β-lactamases (MBLs), extended-spectrum β-lactamases (ESBLs), and AmpC β-lactamases represent key groups within this category of enzymes [5].

This underscores the imperative for clinical microbiologists to identify these β-lactamases producing Enterobacteriaceae through efficient antimicrobial susceptibility testing, employing straightforward and swift screening and confirmatory methodologies. Aligned with the assertion above, this study seeks to examine the occurrence of multidrug-resistant (MDR) Enterobacteriaceae and to conduct phenotypic screening for β-lactamases and carbapenemases among diverse Enterobacteriaceae isolates obtained from clinical samples.

## Materials and methods

Study design, period, and sample size

This was a cross-sectional analytical (laboratory investigation) study, which involved analyzing 156 Enterobacteriaceae isolates from various clinical samples from November 2022 to November 2023 at the Department of Microbiology, Krishna Institute of Medical Sciences, Krishna Hospital and Medical Research Centre, Karad. The investigation followed ethical standards and received approval from the Institutional Ethics Committee, Krishna Institute of Medical Sciences (Deemed to be University), Karad, through protocol number 393/2020-2021.

Sample collection and processing

All the samples received from Krishna Hospital in the Department of Microbiology were included and processed per the standard guidelines. Gram staining was performed to identify the Enterobacteriaceae isolates. The specimens were inoculated on nutrient agar, MacConkey agar, blood agar, and chocolate agar and incubated at 37°C for 24 hours. Isolates were identified based on colony morphology on agar plates, and further biochemical reactions were carried out to confirm the organism [6].

Antimicrobial susceptibility test

Isolates were tested for antimicrobial susceptibility on Mueller-Hinton agar using the Kirby-Bauer disc diffusion method, according to Clinical and Laboratory Standards Institute (2023) guidelines [7]. The antibiotic discs (HiMedia Laboratories Pvt. Ltd., Mumbai, India) used were amikacin (30 µg), ciprofloxacin (5 µg), imipenem (10 µg), meropenem (10 µg), gentamicin (10 µg), levofloxacin (5 µg), tigecycline (15 µg), co-trimoxazole (25 µg), nalidixic acid (30 µg), ampicillin (10 µg), and ceftazidime (30 µg).

Detection of MBL production by imipenem-EDTA combined disk method

The imipenem-ethylenediaminetetraacetic acid (EDTA) combined disc diffusion method was used to detect and confirm MBL producers. The imipenem-EDTA combined disc diffusion test (CDDT) identified MBL producers by comparing inhibition zones of imipenem alone and imipenem+EDTA discs. An increase of 7 mm or more with imipenem-EDTA indicated MBL-producing bacteria [7].

Detection of ESBL production by CDDT

To detect ESBLs, bacterial strains were grown on the Mueller-Hinton agar and tested using the CDDT. The turbidity of the test organisms was adjusted to a 0.5 McFarland standard. A disc with ceftazidime and clavulanic acid was placed 20 mm from a ceftazidime disc, and the plates were incubated at 37°C for 18-24 hours. A 5 mm or greater increase in the inhibition zone around the ceftazidime disc indicated a positive ESBL result [7].

Detection of AmpC β-lactamase production by boronic acid disc test

To identify AmpC β-lactamase producers, the bacterial inoculum was adjusted to a 0.5 McFarland standard and inoculated on Mueller-Hinton agar. Phenylboronic acid was added to cefoxitin discs, placed 20 mm apart on the agar, and incubated at 37°C for 18-24 hours. If the inhibition zone around the cefoxitin+phenylboronic acid disc was 5 mm or more than that around the cefoxitin disc, the organism was considered an AmpC β-lactamase producing bacteria [8].

Statistical analysis

Data were input into an Excel sheet and analyzed with Microsoft Excel (Microsoft Corporation, Redmond, WA). The results were expressed as percentages, and tables were generated to meet different objectives. Table 1 shows the details of the equipment used during the study, including their LOT number and expiration date.

**Table 1:** Details of the equipment used in the study

Equipment	Lot no.	Expiry date
Nutrient agar	0000636975	2029-02
Blood agar base	0000642465	2029-03
MacConkey agar	0000635146	2029-02
Mueller-Hinton agar	0000648002	2029-04
Tryptose phosphate broth	0000505992	2026-10
Buffered glucose broth	0000532471	2025-04
Simmons citrate agar	0000362132	2025-12
Urea agar base	0000125391	2025-05
Triple sugar iron agar medium	0000438981	2025-05
Agar powder bacteriological grade	0000363028	2024-09
Amikacin (30 µg)	0000622313	2025-04
Ciprofloxacin (5 µg)	0000611646	2025-04
Imipenem (10 µg)	0000613280	2024-08
Meropenem (10 µg)	0000598631	2024-04
Gentamicin (10 µg)	0000574414	2025-02
Levofloxacin (5 µg)	0000614765	2024-03
Tigecycline (15 µg)	0000609475	2024-12
Co-trimoxazole (25 µg)	0000556081	2024-05
Nalidixic acid (30 µg)	0000576221	2024-05
Ampicillin (10 µg)	0000612044	2024-09
Ceftazidime (30 µg)	0000565369	2024-03
Cefoxitin (30 µg)	0000440989	2025-10

## Results

From November 2022 to November 2023, the Department of Microbiology, Krishna Institute of Medical Sciences (Deemed to be University), Karad, studied 156 bacterial isolates obtained from patients admitted to various medical, surgical, and intensive care units (ICUs).

The data in Table 2 illustrate the distribution of maximum isolates from various specialized care units. The medicine ICU accounted for 45 isolates (28.8%), followed by the surgery ICU with 40 isolates (25.6%), the neurology ICU with 36 isolates (23.07%), oncology with 22 isolates (14.1%), casualty ICU with six isolates (3.84%), neonatal intensive care unit with four isolates (2.56%), and obstetrics and gynecology with three isolates (1.99%).

**Table 2:** Distribution of Enterobacteriaceae isolates in hospital ICU: intensive care unit; NICU: neonatal intensive care unit; OBGY: obstetrics and gynecology

Department	Number (n)	Percentage (%)
Medicine ICU	45	28.80
Neurology ICU	36	23.07
Oncology	22	14.10
Surgery ICU	40	25.64
Casualty ICU	6	3.84
NICU	4	2.56
OBGY	3	1.99

Of the 156 isolates, the maximum number was from urine (56), followed by tracheal aspirates (36), pus and wound swab (34), sputum (18), blood (11), and cerebrospinal fluid (1) (Table 3).

**Table 3:** Distribution of the Enterobacteriaceae isolates from various clinical specimens CSF: cerebrospinal fluid

Organisms	Urine	Sputum	Blood	CSF	Tracheal aspirate	Pus and wound swab	
							E. coli	26	5	3	1	7	10	
K. pneumoniae	17	12	8	0	24	18	
*E. cloacae* complex	8	1	0	0	5	3	
P. mirabilis	4	0	0	0	0	3	
C. freundii	1	0	0	0	0	0	
Total (n = 156)	56	18	11	1	36	34	

According to the data, out of the 156 gram-negative organism isolates, *Klebsiella pneumoniae* accounted for 79 (50.6%), making it the most common isolate, followed by *E. coli* with 52 isolates (33.3%), *Enterobacter cloacae* complex with 17 isolates (10.8%), *and Proteus mirabilis* with seven isolates (4.4%). The least isolated strain was *Citrobacter freundii, with only* one isolate accounting for 0.9% of the total isolates (Table 4).

**Table 4:** Enterobacteriaceae isolates with percentage

Organism	No. of isolates (n)	Percentage (%)
K. pneumoniae	79	50.6
E. coli	52	33.3
*E. cloacae* complex	17	10.8
P. mirabilis	7	4.4
C. freundii	1	0.9

Among the 156 Enterobacteriaceae isolates, *E. coli *showed maximum sensitivity to meropenem with 25 isolates (48%) and maximum resistance to ampicillin with 41 isolates (78.8%), followed by ciprofloxacin with 40 isolates (76.9%) and gentamicin with 40 isolates (76.9%). *K. pneumoniae* showed maximum sensitivity to levofloxacin with 22 isolates (27.8%) and maximum resistance to imipenem with 69 isolates (87.3%). *E. cloacae* complex showed maximum sensitivity to imipenem with 12 isolates (70.5%) and levofloxacin with 12 isolates (70.5%), and maximum resistance to ceftazidime with 12 isolates (70.5%). *P. mirabilis* showed 100% sensitivity to amikacin, imipenem, meropenem, levofloxacin, and ampicillin and maximum resistance to ciprofloxacin with two isolates (28.5%) and co-trimoxazole with two isolates (28.5%). *C. freundii* showed 100% sensitivity to amikacin, imipenem, meropenem, gentamicin, and levofloxacin (Table 5).

**Table 5:** Antibiotic susceptibility pattern of Enterobacteriaceae isolates S: sensitive; R: resistant

Antibiotic	*E. coli* (n = 52)		*K. pneumoniae* (n = 79)		*E. cloacae* complex (n = 17)		*P. mirabilis* (n = 7)		*C. freundii* (n = 1)	
	S (%)	R (%)	S (%)	R (%)	S (%)	R (%)	S (%)	R (%)	S (%)	R (%)
Amikacin	18 (34.6)	34 (65.3)	11 (13.9)	68 (86)	7 (41.1)	10 (58.8)	7 (100)	0 (0)	1 (100)	0 (0)
Ciprofloxacin	12 (23)	40 (76.9)	16 (20.2)	63 (79.7)	9 (52.9)	8 (47)	5 (71.4)	2 (28.5)	0 (0)	1 (100)
Imipenem	22 (42.3)	30 (57.6)	10 (12.6)	69 (87.3)	12 (70.5)	5 (29.4)	7 (100)	0 (0)	1 (100)	0 (0)
Meropenem	25 (48)	27 (51.9)	12 (15.1)	67 (84.8)	11 (64.7)	6 (35.2)	7 (100)	0 (0)	1 (100)	0 (0)
Gentamicin	12 (23)	40 (76.9)	19 (24)	60 (75.9)	10 (58.8)	7 (41.1)	6 (85.7)	1 (85.7)	1 (100)	0 (0)
Levofloxacin	21 (40.3)	31 (59.6)	22 (27.8)	57 (72.1)	12 (70.5)	5 (29.4)	7 (100)	0 (0)	1 (100)	0 (0)
Tigecycline	13 (25)	39 (75)	11 (13.9)	68 (86)	9 (52.9)	8 (47)	6 (85.7)	1 (85.7)	0 (0)	1 (100)
Co-trimoxazole	15 (28.8)	37 (71.1)	15 (18.9)	64 (81)	8 (47)	9 (52.9)	5 (71.4)	2 (28.5)	0 (0)	1 (100)
Nalidixic acid	14 (26.9)	38 (73)	20 (25.3)	59 (74.6)	12 (70)	5 (29.4)	6 (85.7)	1 (85.7)	0 (0)	1 (100)
Ampicillin	11 (21.1)	41 (78.8)	12 (15.1)	67 (84.8)	7 (41.1)	10 (58.8)	7 (100)	0 (0)	0 (0)	1 (100)
Ceftazidime	16 (30.7)	36 (69.2)	21 (26.5)	58 (73.4)	5 (29.4)	12 (70.5)	6 (85.7)	1 (85.7)	0 (0)	1 (100)

Of the 156 Enterobacteriaceae isolates, *K. pneumoniae* displayed the highest MBL positivity at 55 (69.6%), followed by *E. coli* at 42 (80.7%). *P. mirabilis* 2 (28.5%) showed the lowest percentage (Table 6).

**Table 6:** Distribution of MBL producers among Enterobacteriaceae isolates MBL: metallo-β-lactamase

Organism	MBL producer, n (%)	Non-MBL producer, n (%)
*E. coli* (n = 52)	42 (80.7)	10 (19.3)
*K. pneumoniae* (n = 79)	55 (69.6)	24 (30.4)
*E. cloacae* complex (n = 17)	6 (35.2)	11 (64.8)
*P. mirabilis* (n = 7)	2 (28.5)	5 (71.5)
*C. freundii* (n = 1)	0 (0.0)	1 (100)

Of the 156 Enterobacteriaceae isolates, *K. pneumoniae* demonstrated the highest ESBL production at 32 (40.5%), while *P. mirabilis* showed the lowest at 3 (42.8%) (Table 7).

**Table 7:** Distribution of ESBL producers among Enterobacteriaceae isolates ESBLs: extended spectrum of β-lactamases

Organism	ESBL producer, n (%)	Non-ESBL producer, n (%)
*E. coli* (n = 52)	18 (34.6)	34 (65.4)
*K. pneumoniae* (n = 79)	32 (40.5)	47 (59.5)
*E. cloacae* complex (n = 17)	5 (29.4)	12 (70.6)
*P. mirabilis* (n = 7)	3 (42.8)	4 (57.2)
*C. freundii* (n = 1)	1 (100)	0 (0.0)

*K. pneumoniae* showed a maximum AmpC production of 35 (44.3%), whereas the *E. cloacae *complex showed a minimum of 5 (29.4%) (Table 8).

**Table 8:** Distribution of AmpC producers among Enterobacteriaceae isolates

Organism	AmpC producer, n (%)	Non-AmpC producer, n (%)
*E. coli* (n = 52)	20 (38.4)	32 (61.6)
*K. pneumoniae* (n = 79)	35 (44.3)	44 (55.7)
*E. cloacae* complex (n = 17)	5 (29.4)	12 (70.6)
*P. mirabilis* (n = 7)	0 (0.0)	7 (100)
*C. freundii* (n = 1)	0 (0.0)	1 (100)

Of the 156 Enterobacteriaceae isolates, the percentage of multidrug resistance was 92.6%. Among these multidrug-resistant isolates, *K. pneumoniae* 68 (43.5%) showed maximum resistance, followed by *E. coli* 52 (33.3%) and *E. cloacae* complex 17 (10.8%) (Table 9).

**Table 9:** Multidrug-resistant pattern of Enterobacteriaceae isolates RG0: resistant to zero group of antimicrobials, i.e., sensitive to all group of antimicrobials used; RG1: resistant to one group of antimicrobials; RG2: resistant to two groups of antimicrobials; RG3: resistant to three groups of antimicrobials; RG4: resistant to four groups of antimicrobials; ≥RG5: resistant to greater than or equal to five groups of antimicrobials; MDR: multidrug-resistant

Bacterial isolate	RG0	RG1	RG2	RG3	RG4	≥RG5	MDR (RG3 + RG4 + ≥RG5), n (%)
E. coli	0	1	3	4	7	41	52 (33.3)
K. pneumoniae	1	4	2	4	4	60	68 (43.5)
*E. cloacae* complex	0	0	1	4	5	8	17 (10.8)
P. mirabilis	0	0	0	2	1	4	7 (4.4)
C. freundii	0	0	0	1	0	0	1 (0.6)
Total	0	5	6	15	17	113	145 (92.6)

**Figure 1:**
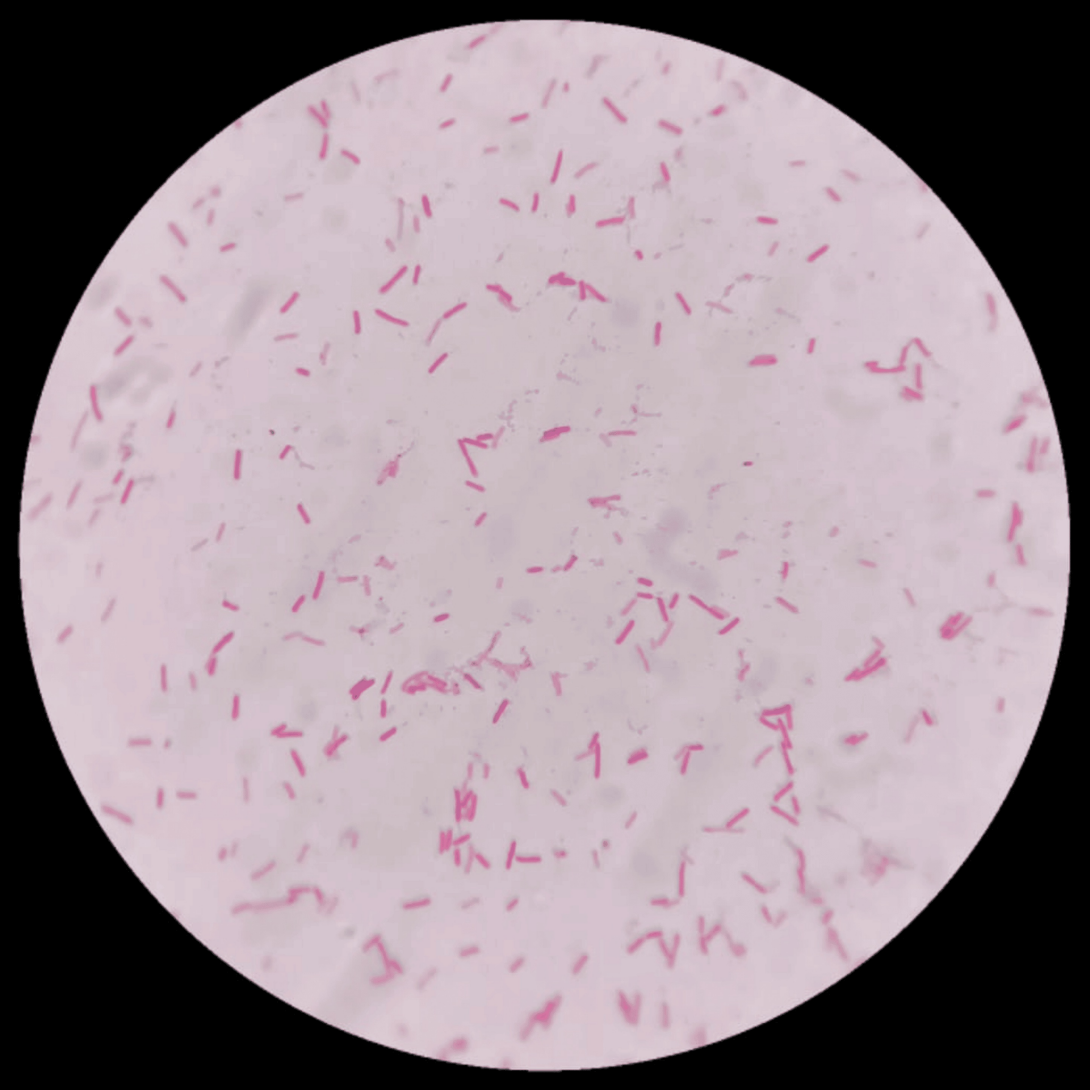
Gram-negative bacilli (100×)

Figure 1 shows gram-negative bacilli at 100× (oil immersion).

**Figure 2:**
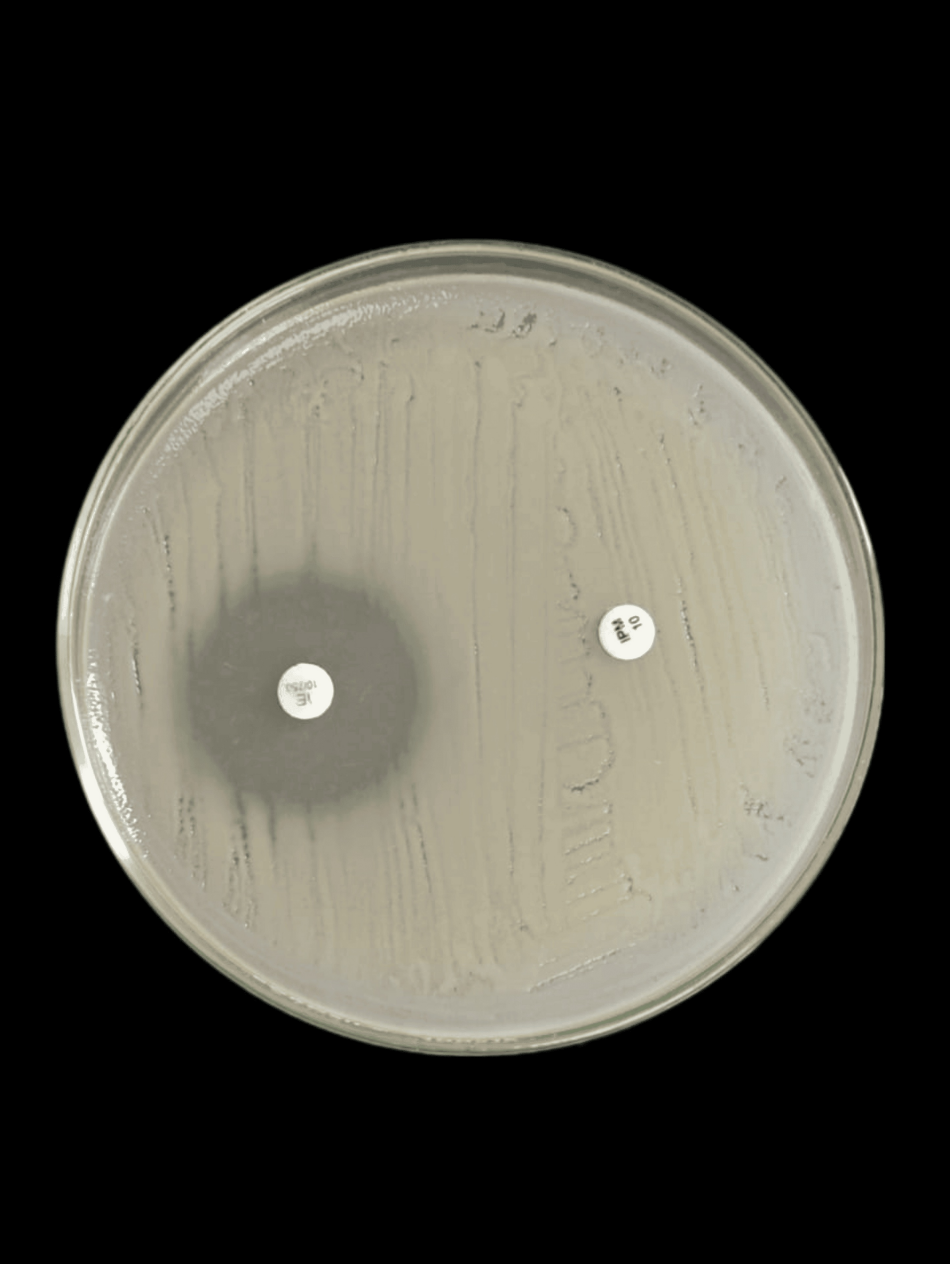
MBL production in K. pneumoniae MBL: metallo-β-lactamase

Figure 2 shows MBL production in *K. pneumoniae*.

**Figure 3:**
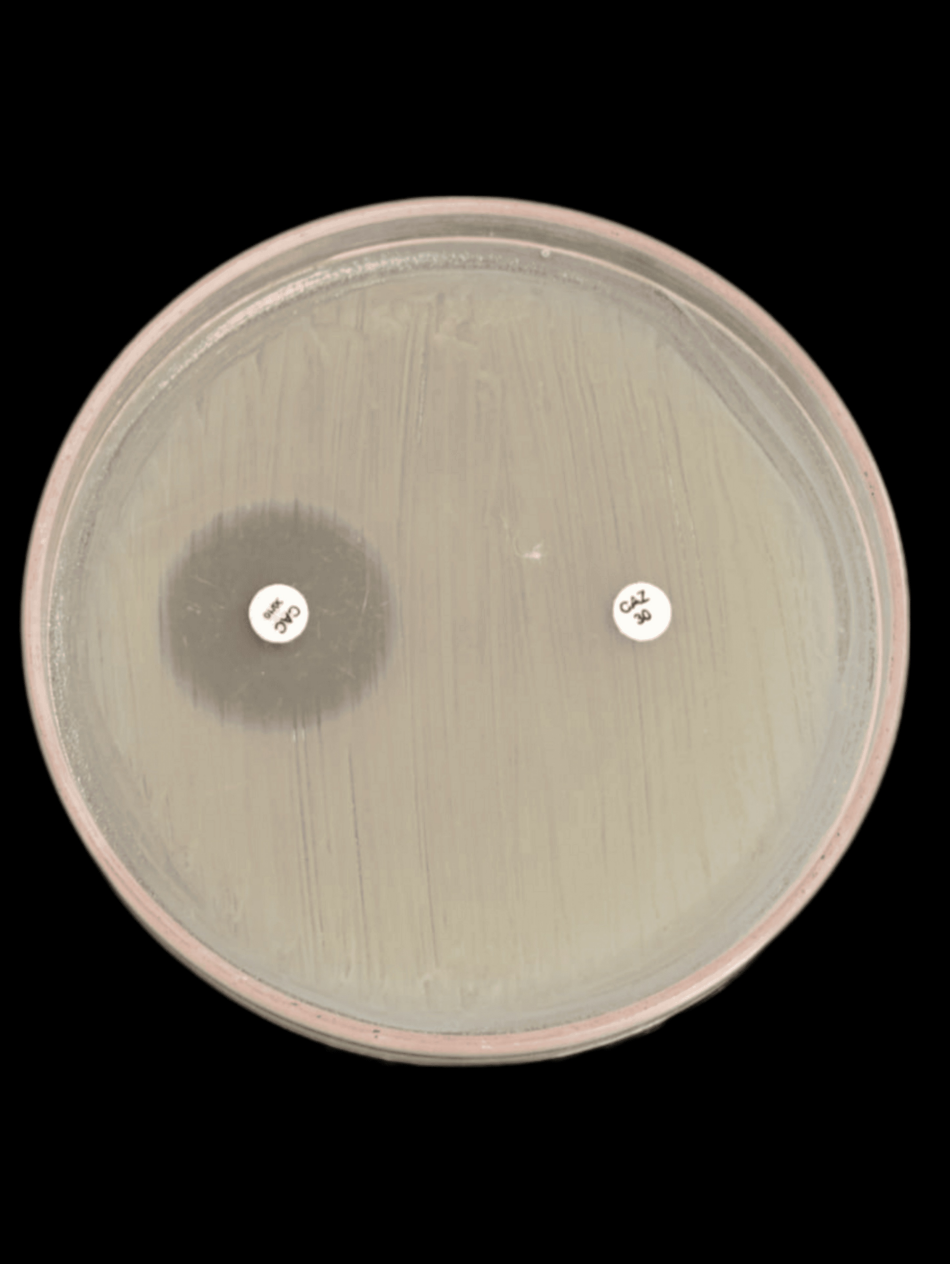
ESBL production in E. coli ESBL: extended-spectrum β-lactamase

Figure 3 shows ESBL production in *E. coli*.

**Figure 4:**
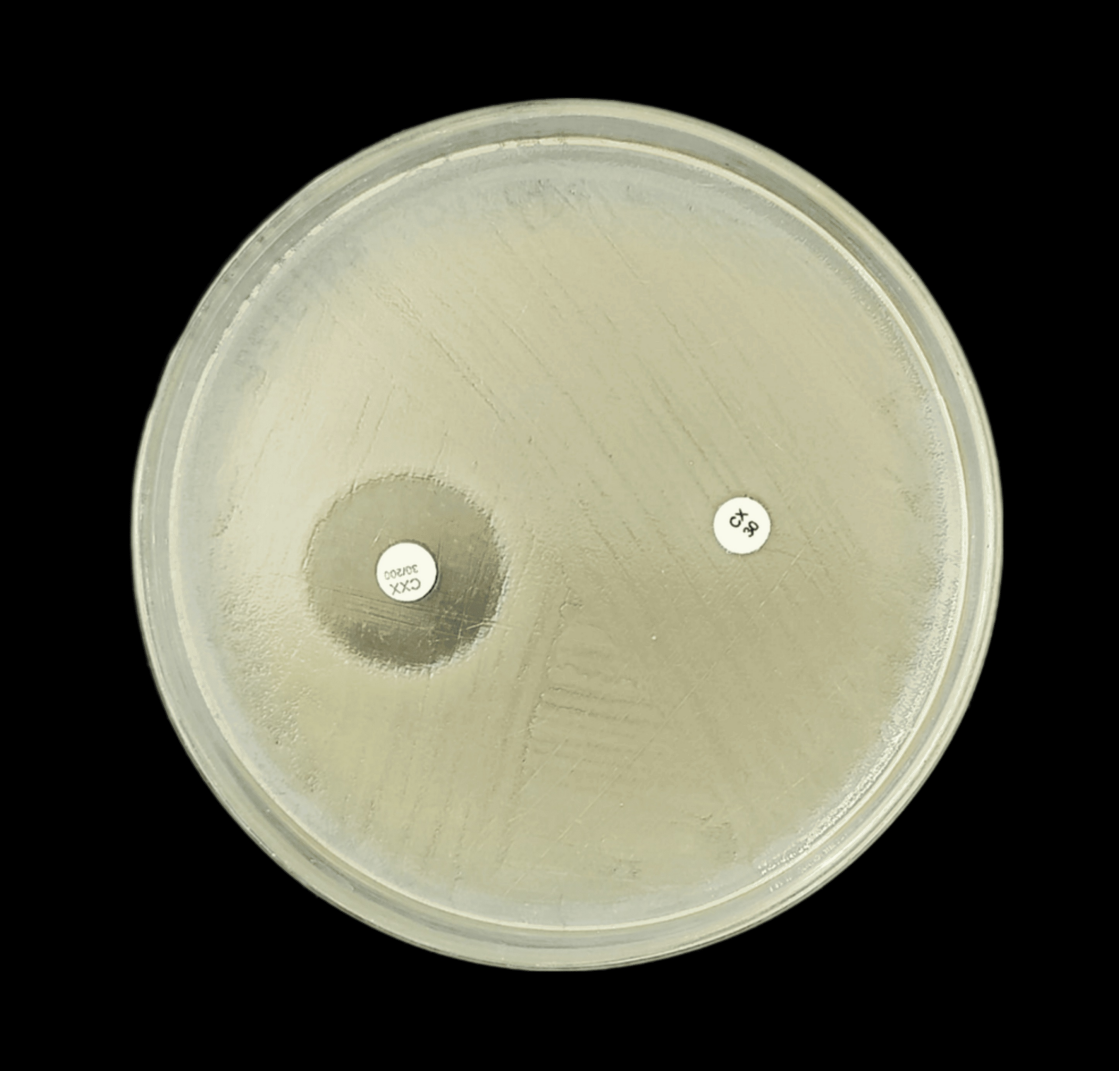
AmpC production in K. pneumoniae

Figure 4 shows AmpC production in *K. pneumoniae*.

## Discussion

Members of Enterobacteriaceae inhabit the gastrointestinal tract in humans and animals, acting as reservoirs of contaminants and pathogens [9]. Global antibiotic resistance is a growing concern due to the widespread and often indiscriminate use of antibiotics in human healthcare, aquaculture, and animal husbandry [10].

Antimicrobial resistance levels vary significantly between healthcare facilities and geographical regions. In Asia, notable geographic disparities in bacterial pathogen resistance exist compared to Western countries [11]. Assessing the accurate burden of antibiotic resistance in India is challenging due to the absence of a mandatory national surveillance system, variations in methodologies for antibiotic susceptibility testing, and the lack of mandatory quality assurance, quality control, and accreditation standards for testing laboratories [12].

As per our study, most isolates were from patients admitted to the medicine ICU (28.8%), followed by the surgery ICU (25.64%). This was well in correlation with the study of Thomas and Sarwat, who reported similar isolation from medicine ICU (27.5%) [13]. The antibiotic resistance pattern revealed that the maximum resistance was to co-trimoxazole (96.3%), ciprofloxacin (88.7%), and ceftazidime (84.6%). Further, the study carried out by Iman et al. showed complete resistance to ceftazidime and minimum resistance to meropenem (19.6%) [14].

Of the 156 Enterobacteriaceae isolates, MBL production was 67.3%, similar to the findings by Swaminathan et al., reporting 74% MBL production [15]. ESBL producers were 67.8%, slightly higher than the study by Sangare et al., which documented 61.8% [16]. In addition, 38.4% were AmpC producers among 156 Enterobacteriaceae isolates. A similar observation was made by Roopashree and Kaup, which showed 37.8% of isolates positive for AmpC production [17]. Over half (92.6%) of the 156 Enterobacteriaceae isolates were MDR. The results of the present study are identical to Fazeli et al., who reported 91.5% MDR [18]. Due to increasing resistance, options for antibiotics become even more limited, such as only colistin and similar drugs remaining effective. However, they also have many different side effects.

One primary limitation of the current study was the failure to investigate the isolates for their MIC breakpoints and analyze them at the molecular level, which would have allowed us to observe the frequency of genes in the isolates.

## Conclusions

The research findings from Western Maharashtra portray ample dispersion of Enterobacteriaceae isolates from a tertiary care hospital. MBL, ESBL, and AmpC prevalence increased significantly in our study. In the present study, MDR bacteria were most commonly isolated from patients admitted to the ICU. ICU patients, who frequently have underlying conditions and use medical devices, are at greater risk of encountering resistant organisms due to high patient turnover and frequent transmission opportunities. Our hospital is surrounded by hills, where most people are engaged in agriculture and animal husbandry. Due to these factors, the presence of many such resistance patterns is evidence of the exploitation of antibiotics in humans and animals. Detecting and understanding β-lactamases is crucial to prevent infections and ensure they do not recur. Additionally, promoting similar studies in specific geographic areas is essential to delineate organism-specific antibiotic susceptibility patterns and β-lactamase production, facilitating effective management and treatment strategies.
